# Depression among lumbar spine surgery patients: Uncovering the untold story

**DOI:** 10.1016/j.xnsj.2025.100846

**Published:** 2026-01-03

**Authors:** Aboubacar Wague, Jennifer M. O’Donnell, Nesa Milan, Alex Youn, Gurbinder Singh, Anna Filley, Avionna Baldwin, Zodina Beiene, Aesha Ajose, Ashraf N. El Naga, David Gendelberg, Sigurd Berven

**Affiliations:** a University of California, San Francisco School of Medicine (UCSF SOM), San Francisco, CA 94143, United States; b Department of Orthopaedic Surgery, University of California San Francisco, San Francisco, CA 94143, United States; c Department of Orthopaedic Surgery, Zuckerberg San Francisco General Hospital, San Francisco, CA 94110, United States

**Keywords:** PROMIS, Depression, Lumbar spine surgery, Patient-reported outcomes, Undiagnosed depression, Psychiatric screening

## Abstract

**Background:**

Depression is highly prevalent among patients with lumbar degenerative disease and is associated with worse postoperative outcomes. However, a significant proportion of affected individuals remain undiagnosed. We aimed to evaluate the utility of PROMIS Depression scores in identifying patients with undiagnosed depression undergoing lumbar spine surgery and to characterize their clinical outcomes relative to patients with diagnosed and no depression.

**Methods:**

This retrospective cohort study included patients undergoing 1- or 2-level lumbar decompression or fusion between March 2019 and November 2021. Patients were stratified into three cohorts: diagnosed depression (PDD), no depression (NDD), and at-risk for undiagnosed depression (ARUD), defined as PROMIS Depression ≥55 without an ICD-10 diagnosis. Patient-reported outcomes were assessed preoperatively and at 6, 12, and 24 months postoperatively. Between group comparisons and baseline-adjusted ANCOVA models were performed, including subgroup analyses by procedure type (fusion vs. decompression) and revision status.

**Results:**

Of 286 ICD-10-negative patients, 24.1% (n=69) met criteria for ARUD. Patient-reported outcome scores across all domains in the ARUD cohort mirrored those of the PDD group and were significantly worse than those of the NDD cohort at all timepoints (p<.001). PROMIS Depression showed a strong correlation with Anxiety (ρ>0.77) and moderate correlations with other domains. No significant difference was observed in outcomes between treated and untreated PDD patients. Older age was associated with reduced likelihood of diagnosis, while substance abuse history, pain clinic enrollment, and retired status predicted higher risk of undiagnosed depression.

**Conclusions:**

PROMIS Depression scores can identify patients with undiagnosed depression who experience similar impairments and postoperative outcomes as those with diagnosed depression. These findings support the routine use of PROMIS Depression as a screening tool to enhance preoperative psychiatric assessment in spine surgery patients.

## Introduction

Lumbar degenerative disease and depression are commonly present together in many patients. It is estimated that approximately 1 in 3 low back pain patients experience depression [[1], [2], [3]]. In patients undergoing surgery for chronic low back pain, depression has been associated with worse disability and pain perception, longer hospital length of stay (LOS), and higher rates of postoperative delirium [4,5]. While some studies have shown that depressive symptoms can improve post-surgery, these patients continue to have worse outcomes compared with those without symptoms of depression preoperatively [6,7].

Current studies indicate that a significant proportion of individuals with depression lack a formal diagnosis [8]. Untreated depression can have profound long-term consequences for quality of life, productivity, and overall health [8,9]. To combat this, physicians may use depression-specific screening tools that can help identify individuals with underlying depression. Such identification allows for appropriate patient referral and improved patient counseling. Among these, the Patient-Reported Outcomes Measurement Information System (PROMIS) consists of a series of surveys that have been validated in lumbar spine patients and are currently used in many clinical practices [10]. Specifically, the PROMIS domain of Depression has been shown to correlate well with formal depression screening tools such as the Patient Health Questionnaire 9-item and 2-item scales (PHQ-9 and PHQ-2) and the 36-Item Short Form Survey Instrument (SF-36) Mental Health scale [11]. Additionally, it has been shown to demonstrate reliability in detecting depression in musculoskeletal patients.

Our objective is to use PROMIS Depression scores to identify individuals with potentially undiagnosed depression and characterize their outcomes relative to patients with diagnosed depression and patients without depression who screen negative on PROMIS Depression. Additionally, we aim to detect risk factors that predict undiagnosed depression in our lumbar spine populations.

## Methods

### Demographic and patient reported outcomes

A retrospective chart review was conducted on a prospectively maintained database. The cohort included consecutive patients who underwent primary or revision 1- or 2-level lumbar decompression or fusion procedures at a single academic institution between March 2019 and November 2021. Patients completed PROMIS Anxiety, Depression, Fatigue, Pain Interference (PI), Physical Function (PF), Sleep Disturbance (SD), and Social Roles (SR) surveys at preoperative intake as well as at 12- and 24-month visits postoperatively. A subset of these patients also completed surveys at 6-month follow-up. Scores for each PROMIS measure are normalized to a mean of 50 based on the United States general healthy population with a standard deviation of 10, with higher scores indicating more of the surveyed entity (eg, depression or anxiety). Exclusion criteria included those with missing prescription data from the electronic medical record (EMR) system, any patients undergoing surgery for a tumor or infection, and patients with a history of depression in remission at the time of surgery, which was determined by a psychiatrist's clinic note to avoid misclassification bias, as they carried an ICD-10 diagnosis for depression but did not exhibit active depressive symptoms at the time of surgery. This study was approved by the hospital’s Institutional Review Board.

A total of 487 patients completed baseline PROMIS surveys, of whom 47 were excluded due to incomplete EMR prescription data (n=28), depression in remission (n=10), or surgery for a primary malignancy (n=9), yielding a final analytic cohort of 440 patients. Follow-up completion rates for PROMIS surveys were 83.9% at 6 months and 100% at 12 and 24 months. Missing PROMIS items were rare (<3%) and were handled using listwise deletion at each timepoint. Patients were routinely contacted via patient portal reminders and telephone follow-up to improve postoperative survey completion. No imputation procedures were applied. Patients were categorized into three groups based on ICD-10 code diagnosis for depression and their baseline PROMIS Depression Scores obtained at intake: 1) positive ICD-10-based diagnosis of depression (PDD), 2) no ICD-10 or PROMIS-based diagnosis of depression (NDD), and 3) at-risk for undiagnosed depression (ARUD). ARUD was defined as patients without an ICD-10-based diagnosis of depression, but who had a PROMIS Depression score ≥ 55 [12].

The PROMIS Depression domain has been validated in spine and musculoskeletal populations and demonstrates high internal consistency and strong correlation with established screening tools, including the PHQ-9 and SF-36 Mental Health subscale [[10], [11], [12]]. A threshold of ≥55 corresponds to approximately 0.5 standard deviations above the population mean and reflects mild depressive symptomatology, consistent with prior literature [12]. ICD-10 codes used to identify depression included F32.0–F32.9 (major depressive disorder, single episode), F33.0–F33.9 (major depressive disorder, recurrent), F34.1 (dysthymic disorder), and F39 (unspecified mood disorder). Codes were identified through a comprehensive review of all available EMR encounters prior to the index surgery.

Demographics and baseline health status were collected, including age, gender, comorbidities, social determinants of health, American Society of Anesthesiology (ASA) score, and prior surgical history. Surgical variables, including surgical invasiveness index (SII), estimated blood loss (EBL), duration of operation, and hospital length of stay (LOS), were also collected. The SII, defined by Mirza et al., is calculated as a sum of scores related to the following six surgical procedures performed at each level: anterior decompression, anterior fusion, anterior instrumentation, posterior decompression, posterior fusion, and posterior instrumentation; the cumulative score ranges from 0 to 48 points, with a higher score indicating greater procedural invasiveness [13]. Both decompression-only and fusion procedures were included to capture the full range of surgical interventions for lumbar degenerative disease. SII was used to quantify procedural complexity and control for differences in surgical magnitude. Patient-reported outcome measures included the Oswestry Disability Index (ODI) and PROMIS PF, PI, Depression, Anxiety, SR, Fatigue, and SD. All data were collected from the EMR and were deidentified.

### Statistical analysis

Primary outcome measures were the between-cohort comparisons of the mean for each PROMIS survey. In addition, change from baseline (Δ) scores for each PROMIS domain and the Oswestry Disability Index (ODI) were calculated at 12 and 24 months. Between group comparisons were further evaluated using analysis of covariance (ANCOVA) models adjusted for baseline score, age, gender, SII, and revision status, with robust standard errors. In addition to the ANCOVA analysis, we referenced previously established minimum clinically important difference (MCID) thresholds to provide clinical context for the magnitude of change in PROMIS and ODI scores. MCID values were used as descriptive indicators of meaningful improvement rather than for statistical responder analysis. Improvements exceeding MCID thresholds were interpreted as clinically relevant but not as the primary inferential outcomes. Comparisons were performed using a simple t-test or ANCOVA when appropriate. Post hoc analysis of ANCOVA was conducted with the Tukey test. Secondary outcome measures were changes in mean PROMIS scores at 12- and 24-month postoperatively. We used the following MCID threshold values for PROMIS measures established by Purvis et al.: PROMIS Anxiety, −4.4; Depression, −6.0; Fatigue, −5.3; PI, −5.4; PROMIS PF, +5.2; SR, +6.0; and SD, −6.5 [14]. For ODI, Copay et. al suggest a MCID value of 12.8 points [15].

Correlations between PROMIS Depression and other PROMIS domain scores were investigated using Spearman's rank correlation. Strength was assessed as “very weak” (0.00 to 0.19), “weak” (0.20 to 0.39), “moderate” (0.40 to 0.59), “strong” (0.60 to 0.79), and “very strong” (0.80 to 1.00) [16]. ANCOVA and t-test were used to obtain p-values for all continuous variables as appropriate, and a chi-square test was performed for categorical variables. All statistical analyses were performed using R software version 4.3.2 with statistical significance defined at p<.05 (R Foundation, Vienna, Austria).

## Results

### Demographic and impact of depression on patient-reported outcome measures

A total of 487 patients completed PROMIS surveys at baseline and postoperatively at 12- and 24-months. From this cohort, 47 patients were excluded: 28 with incomplete prescription data, 10 with a history of depression in remission, and 9 undergoing surgery for a primary diagnosis of cancer. Four hundred forty patients were included in the final cohort. Seventy-five of these patients did not complete surveys at the 6-month timepoint but were included for analysis of outcomes at 12- and 24-months. In total, 286 of 440 patients were ICD-10-negative for depression, and 154 were PDD. No differences between subgroups were observed for age, duration of surgery, postoperative LOS, number of prior spine surgeries, SII, or co-morbidities (Table 1). Of the 365 patients who completed 6-month PROMIS surveys, 231 were ICD-10-negative for depression, and 134 were PDD.

**Table 1:** Clinical characteristics of patients with lumbar degenerative pathology.

Variable	No ICD-10 diagnosis of depression (N=286)	With ICD-10 for depression (N=154)	p-value
Age	64.99 (14.25)	63.32 (13.98)	.236
BMI range (%)			.915
<18.5	2 (0.70%)	2 (1.30%)	
18.5 to <25	84 (29.37%)	45 (29.22%)	
>25 to <30	113 (39.51%)	61 (39.61%)	
>30 to <35	56 (19.58%)	27 (17.53%)	
>35	31 (10.84%)	19 (12.34%)	
Comorbidities, n (%)			
Hypertension	141 (49.30%)	74 (48.05%)	.804
Hyperlipidemia	118 (41.26%)	57 (37.01%)	.388
Diabetes	23 (8.04%)	13 (8.44%)	.875
Chronic heart failure	6 (2.10%)	3 (1.95%)	1
Chronic kidney disease	16 (5.59%)	3 (1.95%)	.121
History of CVA	6 (2.10%)	3 (1.95%)	1
COPD	3 (1.05%)	3 (1.95%)	.426
Liver disease	13 (4.55%)	7 (4.55%)	1
Myocardial infarction	4 (1.40%)	6 (3.90%)	.105
Peptic ulcer disease	2 (0.70%)	2 (1.30%)	.614
Osteoporosis	22 (7.69%)	13 (8.44%)	.926
Substance abuse history	10 (3.50%)	33 (21.43%)	<.001*
Coronary artery disease	17 (5.94%)	11 (7.14%)	.774
History of deep vein thrombosis	6 (2.10%)	10 (6.49%)	.037*
Number of spinal segments fused	1.63 (1.82)	1.79 (2.22)	.454
Estimated blood loss, cc	261.45 (465.41)	263.96 (527.21)	.960
Operation time, minutes	207.73 (138.05)	213.08 (135.75)	.696
ASA score	2.16 (0.51)	2.21 (0.54)	.414
CCI	2.76 (1.87)	2.62 (1.79)	.448
Length of stay, days	2.72 (2.80)	2.88 (2.78)	.568
ICU stay, days	0.20 (1.13)	0.21 (0.95)	.961
Surgical invasiveness index	6.99 (5.96)	7.51 (7.03)	.439
Number prior spine surgeries	0.87 (1.20)	1.10 (1.49)	.110
Revision surgery, n (%)	134 (46.85%)	74 (48.05%)	.889
Chronic opioid use status, 6 months	64 (22.38%)	61 (39.61%)	<.001*

ICD-10, international classification of diseases, 10th revision; BMI, body mass index; CVA, cerebrovascular accident; COPD, chronic pulmonary obstructive disease; ASA, American Society of Anesthesiology; CCI, Charleson comorbidity index; ICU, intensive care unit.

⁎ Statistical significance, p<.05.

Preoperatively, the PDD cohort was associated with significantly worse baseline patient-reported outcomes scores across all survey domains compared to the ICD-10-negative group (Table 2). The PDD cohort continued to have significantly worse outcomes in all domains at 12- and 24-month postoperative follow-up. PROMIS Depression correlated moderately with ODI, PROMIS Fatigue, PF, PI, Sleep Disturbance, and Social Roles, and strongly with PROMIS Anxiety (Table 3).

**Table 2:** Patient-reported health-related quality of life measures in patients with and without an ICD-10 code for depression.

Patient reported outcome measure		ICD-10 negative depression mean (SD)	ICD-10 positive depression mean (SD)	p-value
ODI	Pre op	39.14 (17.03)	46.22 (17.87)	<.001*
	6 month post op	20.03 (17.00)	26.43 (19.63)	.002*
	1 year post op	19.09 (17.26)	25.19 (19.87)	.001*
	2 year post op	20.78 (17.32)	26.65 (21.51)	.004*
PROMIS anxiety	Pre op	52.95 (8.15)	58.73 (8.52)	<.001*
	6 month post op	47.09 (7.77)	54.53 (9.74)	<.001*
	1 year post op	47.68 (8.55)	54.00 (9.35)	<.001*
	2 year post op	48.22 (8.23)	53.72 (9.16)	<.001*
PROMIS depression	Pre op	48.86 (7.58)	54.96 (8.68)	<.001*
	6 month post op	46.03 (7.29)	52.46 (8.75)	<.001*
	1 year post op	46.12 (7.17)	52.45 (8.99)	<.001*
	2 year post op	46.36 (7.46)	52.07 (8.95)	<.001*
PROMIS fatigue	Pre op	52.34 (9.63)	58.71 (9.30)	<.001*
	6 month post op	46.96 (9.65)	53.45 (10.83)	<.001*
	1 year post op	47.34 (9.41)	53.28 (10.54)	<.001*
	2 year post op	48.74 (9.74)	54.30 (9.92)	<.001*
PROMIS pain interference	Pre op	62.94 (7.06)	66.22 (6.61)	<.001*
	6 month post op	54.16 (7.95)	57.71 (8.71)	<.001*
	1 year post op	53.65 (8.90)	57.07 (9.09)	<.001*
	2 year post op	54.07 (8.81)	57.73 (9.92)	<.001*
PROMIS physical function	Pre op	37.07 (6.19)	35.26 (5.05)	.001*
	6 month post op	44.33 (7.82)	42.09 (7.64)	.008*
	1 year post op	45.10 (8.04)	42.55 (8.37)	.002*
	2 year post op	44.39 (8.18)	42.01 (8.69)	.005*
PROMIS sleep disturbance	Pre op	52.81 (8.36)	55.77 (7.35)	<.001*
	6 month post op	48.22 (7.94)	51.65 (8.92)	<.001*
	1 year post op	48.72 (8.11)	51.83 (8.71)	<.001*
	2 year post op	48.40 (8.26)	52.31 (8.26)	<.001*
PROMIS social roles	Pre op	43.65 (8.20)	39.86 (7.46)	<.001*
	6 month post op	51.10 (9.35)	47.02 (8.85)	<.001*
	1 year post op	52.05 (9.53)	48.12 (9.65)	<.001*
	2 year post op	51.03 (9.03)	47.17 (9.83)	<.001*

ODI, Oswestry disability index; PROMIS, patient-reported outcomes measurement information system; ICD-10, international classification of diseases; SD, standard deviation.

⁎ Statistical significance, p<.05.

**Table 3:** Spearman’s rank correlation between PROMIS depression and other PROMs.

	Pre op	6-month post op	1-year post op	2-year post op
ODI	0.432	0.468	0.443	0.447
PROMIS anxiety	0.695	0.797	0.787	0.774
PROMIS fatigue	0.501	0.616	0.593	0.594
PROMIS pain Interference	0.469	0.535	0.453	0.469
PROMIS physical function	−0.346	−0.459	−0.418	−0.434
PROMIS sleep disturbance	0.373	0.514	0.422	0.444
PROMIS social roles	−0.430	−0.547	−0.526	−0.528

All correlations were significant; p<.05.

ODI, Oswestry disability index; PROMIS, patient-reported outcomes measurement information system; PROMs, patient-reported outcome measures.

### Detection of depression, improvement after surgery, and SSRI use

**Fig. 1:**
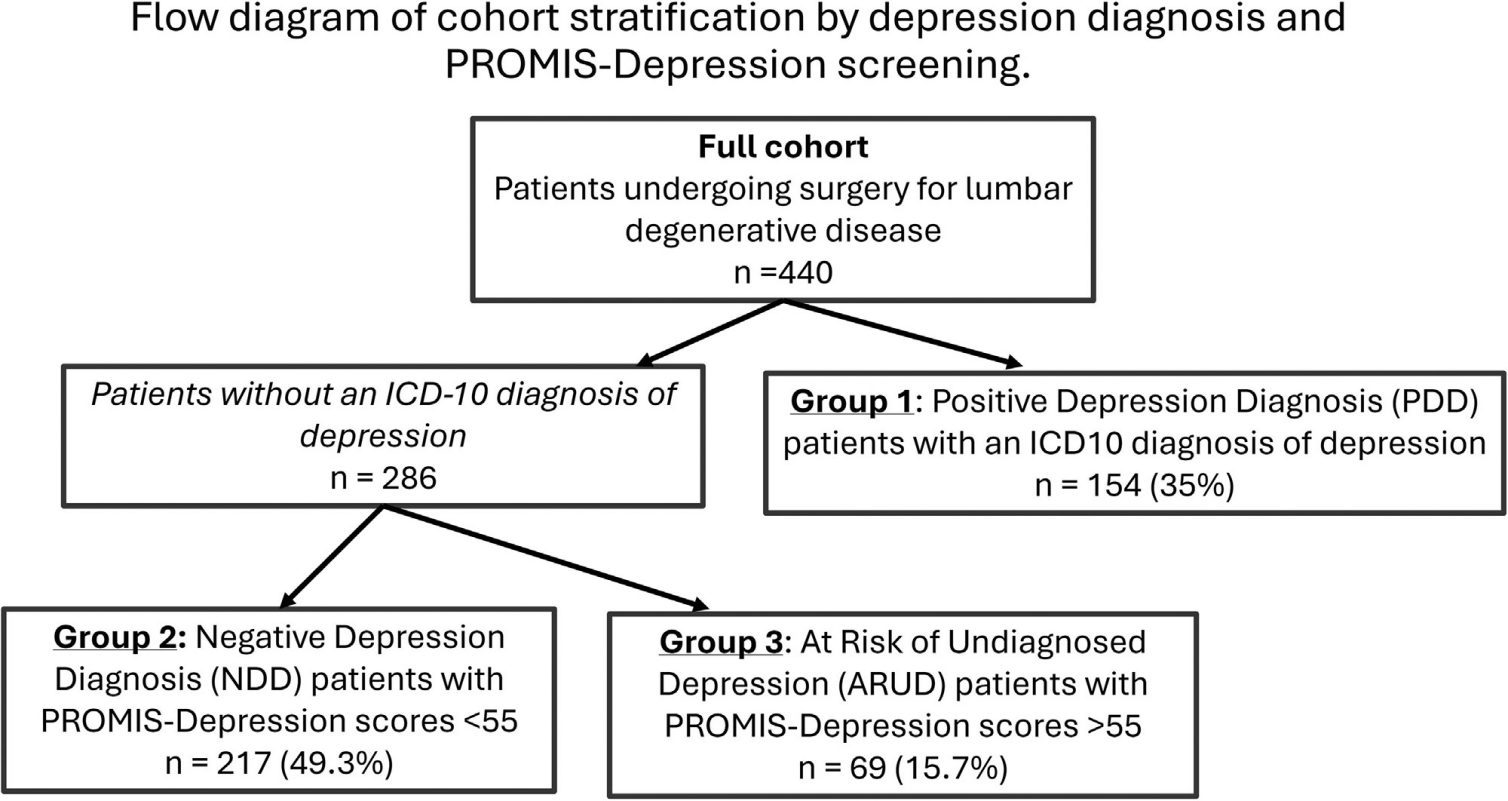
Patients were stratified based on international classification of diseases, 10th Revision (ICD-10) depression diagnoses and Patient-Reported Outcomes Measurement Information System (PROMIS) depression screening into positive depression diagnosis (PDD), negative depression diagnosis (NDD), and at-risk of undiagnosed depression (ARUD) cohorts.

**Fig. 2:**
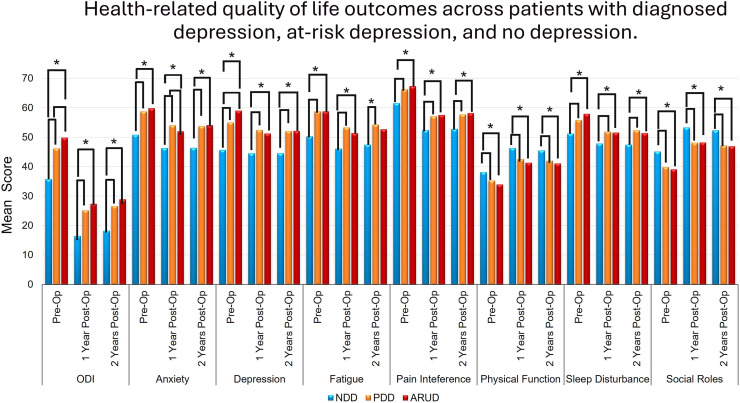
Mean Oswestry disability index (ODI) and patient-reported outcomes measurement information system (PROMIS) domain scores are shown preoperatively and at 1- and 2-year postoperative follow-up for patients with negative depression diagnosis (NDD), positive depression diagnosis PDD), and at-risk of undiagnosed depression (ARUD). *Statistical significance, p<.05.

When the 286 patients in the ICD-10-negative group were further subdivided based on baseline cutoff PROMIS-Depression score of ≥ 55, 69 (24.12%) met criteria for ARUD, and 217 (75.88%) remained in the NDD cohort (Fig. 1). Of the 365 patients who completed 6-month PROMIS surveys, 172 were NDD, 134 were PDD, and 59 were ARUD. The ARUD cohort had worse patient-reported outcomes scores across all domains and all timepoints compared to the NDD cohort (Table 4, Table 5, Fig. 2). Subsequent comparison between PDD and ARUD cohorts revealed no difference in patient-reported outcomes scores across all survey domains and all measured timepoints, except for baseline PROMIS Depression (54.96±8.68 vs. 58.98±3.83, p<.001).

**Table 4:** Outcomes of health-related quality of life measures among patients with and without an ICD-10 diagnosis of depression and those deemed at risk for depression based on elevated PROMIS depression scores.

Patient reported outcome measure		NDD mean (SD)	PDD mean (SD)	ARUD mean (SD)	p-value
ODI	Pre op	35.75 (15.89)	46.22 (17.87)	49.80 (16.18)	<.001*
	6 month post op	16.18 (14.44)†	26.43 (19.63)†	31.19 (18.96)†	<.001*
	1 year post op	16.44 (16.12)†	25.19 (19.87)	27.42 (18.18)	<.001*
	2 years post op	18.20 (16.06)†	26.65 (21.51)	28.90 (18.72)	<.001*
PROMIS anxiety	Pre op	50.78 (7.19)	58.73 (8.52)	59.78 (7.20)	<.001*
	6 month post op	45.38 (6.63)†	54.53 (9.74)	52.06 (8.70)	<.001*
	1 year post op	46.31 (7.81)†	54.00 (9.35)†	52.00 (9.37)†	<.001*
	2 years post op	46.36 (6.94)†	53.72 (9.16)†	54.10 (9.19)†	<.001*
PROMIS depression	Pre op	45.64 (5.30)	54.96 (8.68)	58.98 (3.83)	<.001*
	6 month post op	44.18 (5.53)	52.46 (8.75)	51.41 (8.97)†	<.001*
	1 year post op	44.50 (5.65)	52.45 (8.99)	51.24 (8.88)†	<.001*
	2 years post op	44.52 (5.69)	52.07 (8.95)	52.12 (9.26)†	<.001*
PROMIS fatigue	Pre op	50.30 (9.19)	58.71 (9.30)	58.74 (8.05)	<.001*
	6 month post op	45.53 (9.32)	53.45 (10.83)	51.12 (9.47)	<.001*
	1 year post op	46.08 (9.10)	53.28 (10.54)†	51.32 (9.34)†	<.001*
	2 years post op	47.47 (9.46)	54.30 (9.92)	52.71 (9.59)	<.001*
PROMIS pain interference	Pre op	61.55 (6.99)	66.22 (6.61)	67.32 (5.30)	<.001*
	6 month post op	52.50 (7.75)†	57.71 (8.71)†	58.99 (6.42)†	<.001*
	1 year post op	52.42 (8.84)†	57.07 (9.09)†	57.53 (7.95)†	<.001*
	2 years post op	52.77 (8.56)†	57.73 (9.92)†	58.15 (8.37)†	<.001*
PROMIS physical function	Pre op	38.07 (6.11)	35.26 (5.05)	33.92 (5.36)	<.001*
	6 month post op	45.70 (7.73)†	42.09 (7.64)†	40.38 (6.71)†	<.001*
	1 year post op	46.31 (8.13)†	42.55 (8.37)†	41.32 (6.50)†	<.001*
	2 years post op	45.46 (8.13)†	42.01 (8.69)†	41.05 (7.43)†	<.001*
PROMIS sleep disturbance	Pre op	51.20 (8.03)	55.77 (7.35)	57.88 (7.35)	<.001*
	6 month post op	46.92 (7.47)	51.65 (8.92)	52.01 (8.09)	<.001*
	1 year post op	47.82 (7.95)	51.83 (8.71)	51.55 (8.03)	<.001*
	2 years post op	47.46 (8.08)	52.31 (8.26)	51.38 (8.15)†	<.001*
PROMIS social roles	Pre op	45.10 (7.83)	39.86 (7.46)	39.08 (7.69)	<.001*
	6 month post op	53.09 (8.70)†	47.02 (8.85)†	45.35 (8.85)†	<.001*
	1 year post op	53.28 (9.38)†	48.12 (9.65)†	48.16 (8.99)†	<.001*
	2 years post op	52.35 (8.91)†	47.17 (9.83)†	46.90 (8.17)†	<.001*

PDD, positive diagnosis of depression; NDD, negative diagnosis of depression; ARUD, at risk for undiagnosed depression; ODI, Oswestry disability index; PROMIS, patient-reported outcomes measurement information system; SD, standard deviation.

⁎ Statistical significance, p<.05.

† Minimal clinically important difference, from preoperative to postoperative, within a given cohort.

**Table 5:** Comparison of PROMs between PDD, NDD, and ARUD groups.

Patient reported outcome measure			Mean difference	p-value
ODI	Preop	NDD vs. PDD	−10.47	<.001*
		ARUD vs. PDD	3.58	.300
		ARUD vs. NDD	14.05	<.001*
	6 months post op	NDD vs. PDD	−10.25	<.001*
		ARUD vs. PDD	4.76	.183
		ARUD vs. NDD	15.01	<.001*
	1 year post op	NDD vs. PDD	−8.75	<.001*
		ARUD vs. PDD	2.23	.665
		ARUD vs. NDD	10.98	<.001*
	2 year post op	NDD vs. PDD	−8.45	<.001*
		ARUD vs. PDD	2.25	.680
		ARUD vs. NDD	10.70	<.001*
PROMIS anxiety	Pre op	NDD vs. PDD	−7.95	<.001*
		ARUD vs. PDD	1.05	.614
		ARUD vs. NDD	9.00	<.001*
	6 months post op	NDD vs. PDD	−9.15	<.001*
		ARUD vs. PDD	−2.47	.135
		ARUD vs. NDD	6.69	<.001*
	1 year post op	NDD vs. PDD	−7.69	<.001*
		ARUD vs. PDD	−2.00	.246
		ARUD vs. NDD	5.68	<.001*
	2 year post op	NDD vs. PDD	−7.36	<.001*
		ARUD vs. PDD	0.38	.944
		ARUD vs. NDD	7.74	<.001*
PROMIS depression	Pre op	NDD vs. PDD	−9.32	<.001*
		ARUD vs. PDD	4.02	<.001*
		ARUD vs. NDD	13.34	<.001*
	6 months post op	NDD vs. PDD	−8.28	<.001*
		ARUD vs. PDD	−1.05	.639
		ARUD vs. NDD	7.23	<.001*
	1 year post op	NDD vs. PDD	−7.95	<.001*
		ARUD vs. PDD	−1.21	.509
		ARUD vs. NDD	6.74	<.001*
	2 year post op	NDD vs. PDD	−7.55	<.001*
		ARUD vs. PDD	0.05	.999
		ARUD vs. NDD	7.60	<.001*
PROMIS fatigue	Pre op	NDD vs. PDD	−8.41	<.001*
		ARUD vs. PDD	0.04	1.000
		ARUD vs. NDD	8.45	<.001*
	6 months post op	NDD vs. PDD	−7.92	<.001*
		ARUD vs. PDD	−2.33	.289
		ARUD vs. NDD	5.59	.001*
	1 year post op	NDD vs. PDD	−7.21	<.001*
		ARUD vs. PDD	−1.96	.340
		ARUD vs. NDD	5.24	<.001*
	2 year post op	NDD vs. PDD	−6.82	<.001*
		ARUD vs. PDD	−1.59	.490
		ARUD vs. NDD	5.23	<.001*
PROMIS pain interference	Pre op	NDD vs. PDD	−4.67	<.001*
		ARUD vs. PDD	1.10	.485
		ARUD vs. NDD	5.77	<.001*
	6 months post op	NDD vs. PDD	−5.21	<.001*
		ARUD vs. PDD	1.28	.557
		ARUD vs. NDD	6.49	<.001*
	1 year post op	NDD vs. PDD	−4.65	<.001*
		ARUD vs. PDD	0.46	.931
		ARUD vs. NDD	5.11	<.001*
	2 year post op	NDD vs. PDD	−4.96	<.001*
		ARUD vs. PDD	0.42	.944
		ARUD vs. NDD	5.38	<.001*
PROMIS physical function	Pre op	NDD vs. PDD	2.81	<.001*
		ARUD vs. PDD	−1.33	.233
		ARUD vs. NDD	−4.14	<.001*
	6 months post op	NDD vs. PDD	3.61	<.001*
		ARUD vs. PDD	−1.71	.315
		ARUD vs. NDD	−5.32	<.001*
	1 year post op	NDD vs. PDD	3.75	<.001*
		ARUD vs. PDD	−1.24	.534
		ARUD vs. NDD	−4.99	<.001*
	2 year post op	NDD vs. PDD	3.45	<.001*
		ARUD vs. PDD	−0.96	.700
		ARUD vs. NDD	−4.41	<.001*
PROMIS sleep disturbance	Pre op	NDD vs. PDD	−4.57	<.001*
		ARUD vs. PDD	2.10	.144
		ARUD vs. NDD	6.68	<.001*
	6 months post op	NDD vs. PDD	−4.73	<.001*
		ARUD vs. PDD	0.36	.957
		ARUD vs. NDD	5.09	<.001*
	1 year post op	NDD vs. PDD	−4.02	<.001*
		ARUD vs. PDD	−0.29	.969
		ARUD vs. NDD	3.73	.003*
	2 year post op	NDD vs. PDD	−4.86	<.001*
		ARUD vs. PDD	−0.94	.708
		ARUD vs. NDD	3.92	.002*
PROMIS social roles	Pre op	NDD vs. PDD	5.24	<.001*
		ARUD vs. PDD	−0.78	.761
		ARUD vs. NDD	−6.02	<.001*
	6 months post op	NDD vs. PDD	6.07	<.001*
		ARUD vs. PDD	−1.67	.443
		ARUD vs. NDD	−7.74	<.001*
	1 year post op	NDD vs. PDD	5.17	<.001*
		ARUD vs. PDD	0.05	.999
		ARUD vs. NDD	−5.12	<.001*
	2 year post op	NDD vs. PDD	5.18	<.001*
		ARUD vs. PDD	−0.26	.978
		ARUD vs. NDD	−5.44	<.001*

Post hoc Tukey test of health-related quality of life measures.

PDD, positive diagnosis of depression; NDD, negative diagnosis of depression; ARUD, at risk for undiagnosed depression; ODI, Oswestry disability index; PROMIS, patient-reported outcomes measurement information system; PROMs, patient-reported outcome measures.

⁎ Statistical significance, p<.05.

At 24-month follow-up, the unadjusted mean improvement in PROMIS scores for the ARUD exceeded the MCID in all domains. Improvements in the PDD and NDD cohorts exceeded MCID only for ODI and PROMIS PF, PI, and SR (Table 6). Degree of postoperative improvement was then assessed using baseline-adjusted ANCOVA models controlling for age, gender, revision status, and SII. At 12- and 24-month follow-up, PDD showed significantly less improvement than NDD across all survey domains. While ARUD showed less improvement than NDD in PROMIS PF at 12 months, and in PROMIS Anxiety, PI, PF, and SR at 24 months (Tables 7A and 7B). No significant differences were observed between ARUD and PDD.

**Table 6:** Change in health-related quality of life measures at follow-up.

Patient-reported outcome measure		NDD (SD)	PDD (SD)	ARUD (SD)	p-value
Δ ODI	1-year post op	−19.31 (18.0)†	−21.03 (20.3)†	−22.38 (21.1)†	.456
	2-years post op	−17.55 (18.5)†	−19.57 (21.3)†	−20.90 (24.7)†	.419
Δ PROMIS anxiety	1-year post op	−4.47 (8.36)†	−4.73 (9.03)†	−7.78 (9.73)†	.021*
	2-years post op	−4.42 (7.40)†	−5.01 (9.63)†	−5.68 (9.21)†	.536
Δ PROMIS depression	1-year post op	−1.14 (6.74)	−2.51 (8.25)	−7.74 (8.61)†	<.001*
	2-years post op	−1.12 (7.05)	−2.89 (8.89)	−6.86 (8.72)†	<.001*
Δ PROMIS fatigue	1-year post op	−4.22 (10.2)	−5.42 (10.6)†	−7.43 (10.0)†	.075
	2-years post op	−2.82 (9.81)	−4.41 (10.1)	−6.04 (10.6)†	.051
Δ PROMIS pain interference	1-year post op	−9.12 (9.54)†	−9.15 (8.78)†	−9.79 (9.30)†	.865
	2-years post op	−8.78 (9.49)†	−8.49 (9.76)†	−9.17 (9.79)†	.885
Δ PROMIS physical function	1-year post op	8.24 (8.11)†	7.30 (7.71)†	7.39 (8.30)†	.485
	2-years post op	7.39 (8.47)†	6.75 (8.11)†	7.12 (9.47)†	.774
Δ PROMIS sleep disturbance	1-year post op	−3.38 (7.28)	−3.94 (7.21)	−6.33 (7.67)	.015*
	2-years post op	−3.74 (7.44)	−3.46 (7.49)	−6.50 (9.05)†	.018*
Δ PROMIS social roles	1-year post op	8.18 (9.85)†	8.25 (9.58)†	9.08 (10.9)†	.797
	2-years post op	7.24 (9.94)†	7.30 (10.2)†	7.82 (10.3)†	.914

ΔChange between preoperative score and postoperative score.

PDD, positive diagnosis of depression; NDD, negative diagnosis of depression; ARUD, at risk for undiagnosed depression; ODI, Oswestry disability index; PROMIS, patient-reported outcomes measurement information system; SD, standard deviation.

⁎ Statistical significance, p<.05.

† Minimal clinically important difference, from preoperative to postoperative, within a given cohort.

**Table 7A:** Change from baseline (Δ) and adjusted group comparisons for ODI and PROMIS Scores at 12-month follow-up.

Outcome	Δ 24 mo NDD (Mean ± SD)	Δ 24 mo ARUD (Mean ± SD)	Δ 24 mo PDD (Mean ± SD)	NDD—ARUD (β [95 % CI], p)	NDD—PDD (β [95 % CI], p)	ARUD—PDD (β [95 % CI], p)
ODI	−22.79±16.57†	−18.04±16.39†	−17.91±16.23†	−4.75 [−10.17 to 0.68], .100	−4.88 [−9.01 to −0.75], .016*	−0.13 [−5.62 to 5.36], .998
PROMIS anxiety	−6.64±8.37†	−5.26±8.03†	−2.62±8.09	−1.37 [−4.10 to 1.36], .464	−4.02 [−6.15 to −1.88], <0.001*	−2.64 [−5.30 to 0.01], .052
PROMIS depression	−3.78±7.85	−3.67±7.51	−0.38±7.17	−0.11 [−2.80 to 2.57], .995	−3.40 [−5.40 to −1.41], <0.001*	−3.29 [−5.64 to −0.94], .003*
PROMIS fatigue	−6.74±9.37†	−5.13±9.00	−2.87±9.16	−1.60 [−4.62 to 1.42], .425	−3.86 [−6.25 to −1.48], <0.001*	−2.26 [−5.28 to 0.76], .184
PROMIS pain interference	−10.72±8.49†	−8.24±8.33†	−7.74±8.30†	−2.49 [−5.25 to 0.28], .088	−2.99 [−5.11 to −0.86], .003*	−0.50 [−3.29 to 2.29], .906
PROMIS physical function	9.06±7.41†	6.22±7.34†	6.52±7.27†	2.85 [0.43 to 5.26], .016*	2.55 [0.71 to 4.39], .003*	−0.30 [−2.77 to 2.17], .957
PROMIS sleep disturbance	−4.41±6.97	−4.86±6.89	−3.14±6.80	0.46 [−1.82 to 2.74], .885	−1.27 [−3.00 to 0.46], .197	−1.73 [−4.04 to 0.58], .184
PROMIS social roles	9.81±9.00†	7.48±8.78†	6.64±8.80†	2.32 [−0.59 to 5.24], .146	3.17 [0.92 to 5.42], .003*	0.84 [−2.11 to 3.80], .780

**Table 7B:** Change from baseline (Δ) and adjusted group comparisons for ODI and PROMIS Scores at 24-month follow-up.

Outcome	Δ 24 mo NDD (Mean ± SD)	Δ 24 mo ARUD (Mean ± SD)	Δ 24 mo PDD (Mean ± SD)	NDD—ARUD (β [95 % CI], p)	NDD—PDD (β [95 % CI], p)	ARUD—PDD (β [95 % CI], p)
ODI	−21.30±17.43†	−16.38±17.24†	−16.26±17.07†	−4.92 [−10.62 to 0.79], .107	−5.03 [−9.38 to −0.69], .018*	−0.12 [−5.89 to 5.66], .999
PROMIS anxiety	−6.72±7.96†	−2.98±7.64	−2.84±7.69	−3.74 [−6.34 to −1.14], .002*	−3.88 [−5.91 to −1.86], <.001*	−0.14 [−2.67 to 2.38], .990
PROMIS depression	−4.10±8.15	−2.17±7.80	−0.54±7.44	−1.94 [−4.73 to 0.85], .232	−3.57 [−5.64 to −1.49], <.001*	−1.63 [−4.07 to 0.81], .260
PROMIS fatigue	−5.10±9.24	−3.78±8.87	−2.01±9.03	−1.32 [−4.29 to 1.65], .549	−3.09 [−5.44 to −0.74], .006*	−1.77 [−4.74 to 1.20], .341
PROMIS pain interference	−10.32±8.74†	−7.39±8.57†	−6.98±8.54†	−2.93 [−5.77 to −0.08], .042*	−3.33 [−5.52 to −1.14], .001*	−0.41 [−3.28 to 2.47], .941
PROMIS physical function	8.30±7.64†	5.74±7.56†	5.85±7.49†	2.57 [0.07 to 5.06], .041*	2.46 [0.56 to 4.35], .007*	−0.11 [−2.66 to 2.44], .995
PROMIS Sleep Disturbance	−5.01±7.20	−4.87±7.12	−2.56±7.04	−0.15 [−2.51 to 2.22], .989	−2.45 [−4.24 to −0.66], .004*	−2.31 [−4.69 to 0.08], .061
PROMIS social roles	9.00±8.78†	5.98±8.56	5.52±8.58	3.01 [0.17 to 5.85], .034*	3.48 [1.28 to 5.68], .001*	0.47 [−2.41 to 3.35], .923

PROMIS and ODI Δ values represent mean ± SD (change from baseline post-adjustment for controlled variables). Adjusted group comparisons are β coefficients (95% CI, p) from ANCOVA models controlling for baseline score, age, gender, Surgical Invasiveness Index (SII), and revision status.

PDD, positive diagnosis of depression; NDD, negative diagnosis of depression; ARUD, at risk for undiagnosed depression; ODI, Oswestry disability index; PPROMIS, patient-reported outcomes measurement information system; SD, standard deviation.

⁎ Statistical Significance, p<.05.

† Minimal clinically important difference, from preoperative to postoperative, within a given cohort.

Of the 154 patients with PDD, 106 (68.83%) were receiving treatment with SSRIs, and 72 were not being medically treated for depressive symptoms at the time of surgery. There was no difference in patient-reported outcomes scores between those who were and were not being treated (Table 8).

**Table 8:** Subgroup analysis of patients with PDD, comparison between those who were taking antidepressant medications at the time of surgery and those who were not.

Patient reported outcome measure		Without antidepressant mean (SD)	Receiving antidepressants mean (SD)	p-value
ODI	Pre op	43.88 (19.41)	47.28 (17.12)	.299
	6 month post op	28.74 (21.78)	25.35 (18.56)	.380
	1 year post op	27.04 (22.27)	24.36 (18.73)	.470
	2 years post op	29.83 (24.34)	25.21 (20.06)	.253
PROMIS anxiety	Pre op	57.99 (7.45)	59.06 (8.98)	.438
	6 month post op	55.45 (9.24)	54.10 (9.99)	.440
	1 year post op	54.64 (9.96)	53.71 (9.09)	.580
	2 years post op	54.42 (9.52)	53.40 (9.02)	.534
PROMIS depression	Pre op	53.37 (8.41)	55.68 (8.74)	.121
	6 month post op	52.70 (9.16)	52.35 (8.61)	.835
	1 year post op	52.90 (9.60)	52.25 (8.75)	.689
	2 years post op	52.14 (9.47)	52.04 (8.74)	.954
PROMIS fatigue	Pre op	57.28 (9.19)	59.35 (9.32)	.201
	6 month post op	54.41 (10.95)	53.00 (10.81)	.486
	1 year post op	53.51 (10.84)	53.18 (10.45)	.862
	2 years post op	54.77 (10.19)	54.08 (9.84)	.696
PROMIS pain interference	Pre op	64.92 (7.35)	66.80 (6.18)	.127
	6 month post op	58.19 (9.13)	57.48 (8.55)	.669
	1 year post op	57.68 (9.93)	56.80 (8.72)	.598
	2 years post op	58.15 (11.29)	57.53 (9.29)	.743
PROMIS physical function	Pre op	36.14 (5.13)	34.86 (4.98)	.152
	6 month post op	41.50 (8.23)	42.36 (7.38)	.564
	1 year post op	42.36 (9.11)	42.64 (8.06)	.853
	2 years post op	41.54 (8.97)	42.22 (8.59)	.659
PROMIS sleep disturbance	Pre op	56.14 (7.11)	55.61 (7.49)	.676
	6 month post op	51.87 (9.30)	51.55 (8.79)	.848
	1 year post op	52.23 (9.36)	51.65 (8.44)	.715
	2 years post op	53.36 (9.55)	51.84 (7.61)	.336
PROMIS social roles	Pre op	40.95 (8.41)	39.37 (6.98)	.258
	6 month post op	47.53 (9.37)	46.78 (8.64)	.655
	1 year post op	47.14 (10.80)	48.56 (9.10)	.429
	2 years post op	45.86 (10.53)	47.76 (9.48)	.287

*Statistical significance, p<.05.

PDD, positive diagnosis of depression; ODI, Oswestry disability index; PROMIS, patient-reported outcomes measurement information system; SD, standard deviation.

### Risk factors for undiagnosed depression and surgical subgroup analyses

Additional risk factors, including social determinants of health, which may predict a diagnosis of depression, were collected. Multivariate analysis of these factors, as well as preoperative co-morbidities and other health status indicators, as seen in Supplemental Table 1, revealed that age >60 was associated with decreased likelihood of depression being diagnosed. Additionally, if present, a history of substance abuse, retired work status, and enrollment in the pain clinic were each associated with increased odds of a depression diagnosis (Table 9).

**Table 9:** Multivariate analysis of predictive factors for undiagnosed depression.

	Odds ratio [95% CI]	p-value
Age 60–74	0.502 [0.018, 0.681]	.026*
Age 75+	0.123 [0.015, 0.687]	.027*
Retired work status	2.732 [1.068, 7.143]	.037*
Pain clinic patient	3.175 [1.182, 10.204]	.033*
Substance abuse history	4.032 [1.274, 18.182]	.034*

CI, confidence interval.

⁎ Statistical significance, p<.05.

We compared outcomes between patients undergoing decompression and fusion procedures using baseline-adjusted ANCOVA models controlling for age, gender, and revision status. Patients undergoing fusion were observed to have improved scores in PROMIS Anxiety, Depression, and Fatigue at 24-month follow-up, though these differences were not clinically meaningful (Supplemental Table 2).

We conducted a subgroup analysis comparing outcomes between primary and revision lumbar procedures. In baseline-adjusted ANCOVA models controlling for age, gender, procedure type, and SII, revision status was a significant predictor of worse outcomes across all survey domains and timepoints with the exception of PROMIS Sleep Disturbance and 24-month follow-up of PROMIS Anxiety (Supplemental Table 3).

## Discussion

The purpose of this study was to evaluate the influence of preoperative depression on PROMs following lumbar spine surgery and to validate the utility of the PROMIS Depression survey on identifying patients with undiagnosed depression in this cohort. Our findings support the utility of PROMIS Depression scores as a screening tool for depression to aid spine surgeons in treating one of the most devastating problems and concerns in adult surgical patients [11]. We found that at all measured timepoints, patients determined to be at risk of undiagnosed depression had almost identical outcomes to those who had been clinically diagnosed with depression. PROMIS Depression scores also correlated well with other PROMIS surveys, insinuating that the severity of depressive symptoms correlates with poorer outcomes in function, pain, and quality of life.

Depression has an increasing prevalence in the United States and is commonly associated with degenerative lumbar disease and chronic back pain [[17], [18], [19], [20], [21]]. While it is known that depression is more common in spine patients as compared to the general population, this study highlights that the proportion of spine patients suffering from depression may be underestimated [22]. While 30% of our initial cohort was diagnosed with depression based on ICD10 codes, when including elevated preoperative PROMIS Depression scores, this number increased to 47%. We also identified age >75 and BMI 30–35 as risk factors that increased the odds of patients being at risk for, rather than being diagnosed with, depression. Previous studies have reported correlations between depression and a high BMI, which can be ameliorated with weight loss, as well as increased age [23,24]. Interestingly, we found that patients with a history of substance abuse were more likely to have a clinical diagnosis of depression when compared to the at-risk population. The presence of another psychiatric comorbidity for which treatment was sought provides an opportunity for a comprehensive psychiatric evaluation during which other underlying psychiatric issues, such as depression, can be identified [25,26].

There is a strong link between chronic pain and depression, with the understanding that these two variables are bidirectional [27]. For example, depression has been linked with pain hypersensitivity, supported by our observation that chronic low back pain patients with depression tended to have worse baseline measures of ODI and PROMIS pain interference scores than those without depression [28]. Postoperative relief of low back pain can, in turn, lead to improvement of depressive symptoms, as seen in our study; however, overall outcomes in these patients are still worse in comparison to patients who did not have depression at baseline. These results correspond with previous studies that have shown similar findings [6,29].

Beyond its correlation with pain, depression has been linked to decreased physical function. Studies have shown that amelioration of depression and anxiety is associated with improvement in physical function. Illustrating the bidirectional nature of these relationships, improvement in physical function, similar to the effects of surgery, has been shown to improve depressive symptoms, though to a lesser degree [30]. In our study, we found that baseline PROMIS physical function scores were significantly lower in the PDD and ARUD cohorts when compared to NDD and improved significantly after surgical intervention. While physical function improved, there still may be a residual impact due to the presence of depression, as evidenced by a study conducted by Cenzer et al [31]. Future work will be necessary to explore how various treatment options for depression may impact physical function post-surgery.

Sleep disturbance, fatigue, and diminished social roles are well-known symptoms of, and part of the diagnostic criteria for, depression. Even if depressive symptoms resolve, fatigue and sleep disturbance may persist [32]. These factors may require specific focus to improve patient experience. While social roles are multifaceted and nuanced, the PROMIS SR survey provides a clear metric of one’s satisfaction in this domain of life. Our results demonstrate that satisfaction in social roles inversely correlates with depression. Previous investigations have demonstrated that low levels of social connectedness correlate with higher rates of symptomatic depression and decreased quality of life [33,34]. Future studies should investigate specific aspects of social roles, such as time spent on leisure activities or satisfaction with the ability to do things for family, and their association with depression in our spine population.

Although our study did not detect a difference in patient-reported outcomes scores for depressed patients who were taking antidepressants, a previous study by Cochrane et al. has demonstrated the utility of these medications for improving the PROMIS PI and PF [35]. Furthermore, pretreatment of depression with antidepressants before surgery has been shown to lead to similar outcomes between patients with and without depression in patient-reported outcomes scores measuring pain and functional disability in cervical spine patients [36]. More investigation is needed to determine how these medications may impact other patient-reported outcomes scores, such as social roles, sleep disturbance, and fatigue.

Interventions to treat depression preoperatively in spine surgery patients have also shown promise for reducing readmissions and improving outcomes postoperatively. In a prospective cohort of 140 patients undergoing anterior cervical discectomy and fusion surgery, Elsamadicy et al. showed that preoperatively, treatment of depression resulted in improved patients’ perception of postoperative pain and functional disability [36]. Underscoring the importance of depression on surgical outcomes, the United States Preventive Services Task Force recommends that all patients undergoing spinal surgery go through presurgical psychological screening [37]. However, despite this recommendation, a recent survey of 340 spine surgeons found that only a minority of participating surgeons screened for depression or anxiety preoperatively [37].

Therefore, there remains a need for more widespread attention to preoperative screening and treatment of psychiatric comorbidities such as depression and anxiety in patients undergoing spine surgery. Patients undergoing revision spine surgery often face longer recovery times, greater surgical complexity, and higher rates of persistent pain, which have been linked to worse psychological outcomes. Our study identified revision status as a statistically significant predictor of PROMIS outcomes. Additional research is required to assess if this contributes to a clinically meaningful difference, as long-term outcomes may remain comparable to primary surgeries.

Although this study benefits from a prospectively maintained database and a unique degree of granularity in assessing depression, including identification of undiagnosed depression as a relevant clinical subgroup, several limitations should be acknowledged. The retrospective design introduces potential selection bias and limits causal inference. While decompression and fusion surgeries differ in invasiveness and recovery and lead to statistically significant differences in patient-reported outcomes, no clinically significant differences were observed between these subgroups. However, larger studies may allow for stratified analysis by surgical type.

Additionally, unmeasured confounders such as coping mechanisms, therapy participation, or non-pharmacologic interventions may have influenced outcomes. These findings emphasize the importance of systematic preoperative mental health screening in spine surgery. Integrating PROMIS Depression into standard preoperative assessment and EMR workflows may allow earlier identification and intervention for patients with undiagnosed depression, thereby improving perioperative care, patient counseling, and long-term recovery outcomes. Broader implementation of these screening tools can also inform institutional quality improvement efforts and policy initiatives aimed at integrating psychiatric evaluation into surgical practice.

## Conclusion

PROMIS Depression scores can identify patients with undiagnosed depression undergoing lumbar spine surgery, who exhibit similar pre- and postoperative impairments to those with a formal diagnosis of depression. Routine incorporation of PROMIS Depression into preoperative assessment may help clinicians recognize at-risk patients and optimize perioperative management. Although procedural type did not significantly affect outcomes in this cohort, these factors may influence postoperative recovery and merit investigation in larger, prospective studies.

## Declarations of competing interests

One or more of the authors declare financial or professional relationships on ICMJE-NASSJ disclosure forms.

## Appendix Supplementary materials

Image, application
1

Image, application
2

Image, application
3
