# Supplementary material for: Depression among lumbar spine surgery patients: Uncovering the untold story
Source: N Am Spine Soc J. 2026 Jan 3;25:100846. doi: 10.1016/j.xnsj.2025.100846 (PMC12870787; doi:10.1016/j.xnsj.2025.100846)
Supplement: Supplementary file 1 [file mmc1.docx]

Supplementary Table 1: Comparison of Clinical Characteristics between PDD, NDD, and ARUD groups

| **Variable** | **NDD** | **PDD** | **ARUD** | **p-value** |
| --- | --- | --- | --- | --- |
| Age | 64.94 (14.98) | 63.32 (13.98) | 65.14 (11.77) | 0.497 |
| BMI Range (%) |  |  |  | 0.952 |
| <18.5 | 2 (0.92%) | 2 (1.30%) | 0 (0.00%) |  |
| 18.5 to <25 | 64 (29.49%) | 45 (29.22%) | 20 (28.99%) |  |
| >25 to <30 | 88 (40.55%) | 61 (39.61%) | 25 (36.23%) |  |
| >30 - <35 | 38 (17.51%) | 27 (17.53%) | 18 (26.09%) |  |
| >35 | 25 (11.52%) | 19 (12.34%) | 6 (8.70%) |  |
| Comorbidities, n (%) |  |  |  |  |
| Hypertension | 28.01 (5.62) | 28.17 (6.62) | 27.93 (5.10) | 0.934 |
| Hyperlipidemia | 106 (48.85%) | 74 (48.05%) | 35 (50.72%) | 0.425 |
| Diabetes | 93 (42.86%) | 57 (37.01%) | 25 (36.23%) | 0.729 |
| Chronic Heart Failure | 19 (8.76%) | 13 (8.44%) | 4 (5.80%) | 1.00 |
| Chronic Kidney Disease | 5 (2.30%) | 3 (1.95%) | 1 (1.45%) | 0.178 |
| History of CVA | 12 (5.53%) | 3 (1.95%) | 4 (5.80%) | 0.306 |
| COPD | 3 (1.38%) | 3 (1.95%) | 3 (4.35%) | 0.751 |
| Liver Disease | 3 (1.38%) | 3 (1.95%) | 0 (0.00%) | 1.00 |
| Myocardial Infarction | 10 (4.61%) | 7 (4.55%) | 3 (4.35%) | 0.22 |
| Peptic Ulcer Disease | 4 (1.84%) | 6 (3.90%) | 0 (0.00%) | 0.491 |
| Osteoporosis | 1 (0.46%) | 2 (1.30%) | 1 (1.45%) | 0.904 |
| Substance Abuse History | 16 (7.37%) | 13 (8.44%) | 6 (8.70%) | <0.001* |
| Coronary Artery Disease | 7 (3.23%) | 33 (21.43%) | 3 (4.35%) | 0.931 |
| History of Deep Vein Thrombosis | 13 (5.99%) | 11 (7.14%) | 4 (5.80%) | 0.080 |
| Number of spinal segments fused | 5 (2.30%) | 10 (6.49%) | 1 (1.45%) | 0.729 |
| Estimated Blood Loss, cc | 1.63 (1.77) | 1.79 (2.22) | 1.64 (1.98) | 0.990 |
| Operation Time, minutes | 259.35 (445.31) | 263.96 (527.21) | 268.04 (527.14) | 0.926 |
| ASA score | 208.00 (138.18) | 213.08 (135.75) | 206.91 (138.65) | 0.703 |
| CCI | 2.17 (0.50) | 2.21 (0.54) | 2.16 (0.53) | 0.735 |
| Length of Stay, days | 2.77 (1.83) | 2.62 (1.79) | 2.71 (2.03) | 0.545 |
| ICU Stay, days | 2.64 (2.80) | 2.88 (2.78) | 3.00 (2.78) | 0.927 |
| Surgical Invasiveness Index | 0.22 (1.23) | 0.21 (0.95) | 0.16 (0.72) | 0.575 |
| Number Prior Spine Surgeries | 6.85 (5.83) | 7.51 (7.03) | 7.43 (6.36) | 0.225 |
| Revision surgery, n (%) | 0.86 (1.22) | 1.10 (1.49) | 0.91 (1.15) | 0.421 |
| Chronic opioid use status, 6 months | 97 (44.70%) | 74 (48.05%) | 37 (53.62%) | <0.001* |

*= Statistical Significance, p<0.05

ICD-10, International Classification of Diseases, 10th Revision; BMI, Body Mass Index; CVA, Cerebrovascular accident; COPD, Chronic Pulmonary Obstructive Disease; ASA, American Society of Anesthesiology; CCI, Charleson Comorbidity Index; ICU, intensive care unit
