# Supplementary material for: Depression among lumbar spine surgery patients: Uncovering the untold story
Source: N Am Spine Soc J. 2026 Jan 3;25:100846. doi: 10.1016/j.xnsj.2025.100846 (PMC12870787; doi:10.1016/j.xnsj.2025.100846)
Supplement: Supplementary file 2 [file mmc2.docx]

**Supplementary Table 2.** Baseline adjusted comparison of 12- and 24- month ODI and PROMIS outcomes between decompression and fusion procedures.

| **Outcome** | **Decompression**  **N= 204** | **Fusion**  **N=236** | **β (Decompression - Fusion)** | **95% CI** | **p-value** |
| --- | --- | --- | --- | --- | --- |
| ODI  (12 months) | 20.73 ± 18.91 | 21.77 ± 17.92 | -1.04 | [-4.13 to 2.06] | 0.510 |
| ODI  (24 months) | 22.08 ± 19.34 | 23.54 ± 18.74 | -1.46 | [-4.72 to 1.80] | 0.378 |
| PROMIS Anxiety (12 months) | 50.71 ± 9.40 | 49.34 ± 9.26 | 1.37 | [-0.15 to 2.89] | 0.077 |
| PROMIS Anxiety (24 months) | 51.28 ± 8.91 | 49.29 ± 8.94 | 1.99 | [0.54 to 3.43] | 0.007* |
| PROMIS Depression  (12 months) | 48.95 ± 8.77 | 47.99 ± 8.09 | 0.96 | [-0.37 to 2.28] | 0.155 |
| PROMIS Depression  (24 months) | 49.25 ± 8.60 | 47.78 ± 8.30 | 1.47 | [0.11 to 2.84] | 0.034* |
| PROMIS Fatigue  (12 months) | 50.08 ± 10.75 | 48.91 ± 9.75 | 1.17 | [-0.55 to 2.89] | 0.182 |
| PROMIS Fatigue  (24 months) | 52.05 ± 10.76 | 49.67 ± 9.54 | 2.38 | [0.71 to 4.06] | 0.005* |
| PROMIS Pain Interference  (12 months) | 54.32 ± 9.38 | 55.23 ± 8.82 | -0.91 | [-2.49 to 0.67] | 0.258 |
| PROMIS Pain Interference  (24 months) | 55.19 ± 9.97 | 55.60 ± 8.79 | -0.42 | [-2.05 to 1.22] | 0.619 |
| PROMIS Physical Function  (12 months) | 44.56 ± 8.38 | 43.80 ± 8.04 | 0.76 | [-0.64 to 2.17] | 0.286 |
| PROMIS Physical Function  (24 months) | 44.08 ± 8.77 | 42.94 ± 7.97 | 1.13 | [-0.31 to 2.58] | 0.124 |
| PROMIS Sleep Disturbance  (12 months) | 49.95 ± 8.63 | 49.70 ± 8.31 | 0.25 | [-1.04 to 1.55] | 0.703 |
| PROMIS Sleep Disturbance  (24 months) | 50.25 ± 8.63 | 49.29 ± 8.32 | 0.96 | [-0.39 to 2.31] | 0.162 |
| PROMIS Social Roles  (12 months) | 51.11 ± 9.68 | 50.25 ± 9.79 | 0.86 | [-0.82 to 2.54] | 0.314 |
| PROMIS Social Roles  (24 months) | 49.71 ± 10.13 | 49.53 ± 8.90 | 0.18 | [-1.46 to 1.83] | 0.828 |

Note: Model adjusted for baseline score, age, gender, and revision status. β represents the mean difference in postoperative scores (Decompression minus Fusion).

*= Statistical Significance, p<0.05

ODI, Oswestry Disability Index; Patient-Reported Outcomes Measurement Information System, PROMIS; CI, Confidence Interval
