# Supplementary material for: Depression among lumbar spine surgery patients: Uncovering the untold story
Source: N Am Spine Soc J. 2026 Jan 3;25:100846. doi: 10.1016/j.xnsj.2025.100846 (PMC12870787; doi:10.1016/j.xnsj.2025.100846)
Supplement: Supplementary file 3 [file mmc3.docx]

**Supplementary Table 3.** Baseline-adjusted comparison of 12- and 24-month PROMIS and ODI outcomes between primary and revision surgeries.

| **Outcome** | **Primary (N=232)** | **Revision**  **(N= 208)** | **β**  **(Primary - Revision)** | **95% CI** | **p-value** |
| --- | --- | --- | --- | --- | --- |
| ODI (12 months) | 17.78 ± 16.05 | 24.81 ± 19.52 | -7.02 | [-10.14 to -3.91] | <0.001* |
| ODI (24 months) | 19.33 ± 17.05 | 26.40 ± 19.82 | -7.07 | [-10.34 to -3.79] | <0.001* |
| PROMIS Anxiety  (12 months) | 48.64 ± 8.55 | 51.30 ± 9.86 | -2.65 | [-4.17 to -1.13] | 0.001* |
| PROMIS Anxiety  (24 months) | 49.52 ± 8.45 | 50.89 ± 9.39 | -1.36 | [-2.81 to 0.09] | 0.065 |
| PROMIS Depression  (12 months) | 47.25 ± 7.59 | 49.63 ± 9.06 | -2.38 | [-3.69 to -1.06] | <0.001* |
| PROMIS Depression  (24 months) | 47.39 ± 7.61 | 49.53 ± 9.18 | -2.14 | [-3.50 to -0.78] | 0.002* |
| PROMIS Fatigue  (12 months) | 48.24 ± 9.54 | 50.66 ± 10.64 | -2.41 | [-4.14 to -0.69] | 0.006* |
| PROMIS Fatigue  (24 months) | 49.60 ± 9.39 | 51.93 ± 10.66 | -2.33 | [-4.02 to -0.64] | 0.007* |
| PROMIS Pain Interference (12 months) | 53.42 ± 8.39 | 56.20 ± 9.42 | -2.78 | [-4.37 to -1.19] | 0.001* |
| PROMIS Pain Interference (24 months) | 53.60 ± 9.32 | 57.21 ± 8.74 | -3.61 | [-5.25 to -1.97] | <0.001* |
| PROMIS Physical Function (12 months) | 45.28 ± 7.90 | 43.02 ± 8.25 | 2.26 | [0.86 to 3.66] | 0.002* |
| PROMIS Physical Function (24 months) | 44.67 ± 8.41 | 42.27 ± 8.04 | 2.40 | [0.96 to 3.84] | 0.001* |
| PROMIS Sleep Disturbance (12 months) | 49.27 ± 7.56 | 50.37 ± 9.20 | -1.09 | [-2.39 to 0.21] | 0.099 |
| PROMIS Sleep Disturbance (24 months) | 49.59 ± 7.49 | 49.86 ± 9.36 | -0.27 | [-1.63 to 1.08] | 0.694 |
| PROMIS Social Roles  (12 months) | 52.18 ± 8.95 | 49.12 ± 10.14 | 3.06 | [1.38 to 4.74] | <0.001* |
| PROMIS Social Roles  (24 months) | 51.33 ± 9.00 | 47.90 ± 9.44 | 3.43 | [1.79 to 5.07] | <0.001* |

Model adjusted for baseline score, age, gender, procedure type, and SII.

*= Statistical Significance, p<0.05

ODI, Oswestry Disability Index; Patient-Reported Outcomes Measurement Information System, PROMIS; CI, Confidence Interval, SII, Surgical Invasiveness Index
